# Getting in Shape: Updates in Exercise Anaphylaxis

**DOI:** 10.1007/s11882-024-01176-4

**Published:** 2024-09-19

**Authors:** Annette Carlisle, Jay Adam Lieberman

**Affiliations:** Department of Pediatrics, Division of Pulmonology, Allergy & Immunology, University of Tennessee Health Science Center, LeBonheur Children’s Hospital, 51 N. Dunlap Street, Suite 400, Memphis, TN 38105 USA

**Keywords:** Anaphylaxis, Exercise, Food-induced, Wheat allergy, Cofactor

## Abstract

**Purpose of Review:**

Exercise induced anaphylaxis (EIA) can be difficult to diagnose due to the interplay of co-factors on clinical presentation and the lack of standardized, confirmatory testing.

**Recent Findings:**

EIA has been historically categorized as either food-independent or food-dependent. However, recent literature has suggested that perhaps EIA is more complex given the relationship between not only food on EIA but other various co-factors such as medications and alcohol ingestion that are either required to elicit symptoms in EIA or make symptoms worse.

**Summary:**

For the practicing clinician, understanding how these co-factors can be implicated in EIA can enable one to take a more personalized approach in treating patients with EIA and thus improve quality of life for patients.

## Introduction

Exercise induced anaphylaxis (EIA) is described as anaphylaxis induced by physical exertion [1]. EIA was first described in 1979 by Maulitz et al. when they described a delayed hypersensitivity reaction to shellfish precipitated by jogging in a patient with recurrent anaphylaxis [2]. Since its first description, EIA has been reported world-wide [3–6] with an estimated prevalence of 2.3-5% of all cases of anaphylaxis [7, 8]. It can be categorized as food independent or food dependent, based on whether ingestion to a sensitized food prior to exercise is required to elicit symptoms [9]. Due to various co-factors that can be required for symptoms or determine severity of symptoms (e.g., alcohol ingestion, use of medications, hormonal changes, environmental changes), and the lack of easy standardized confirmatory testing, EIA can be difficult to diagnose. In this article, we aim to provide the practicing clinician up to date information on several aspects of exercise induced anaphylaxis, including updated terminology, diagnostic strategies, differential diagnoses, and management strategies.

## Defining Anaphylaxis

To diagnose EIA, one must be able to make a clear diagnosis of anaphylaxis first. Unfortunately, this is not always easy. Since the creation of the term by Paul Portier and Charles Richet over 100 years ago [10], defining anaphylaxis has been subject to much debate, which in part is due to our lack of a good diagnostic biomarker or test to confirm the diagnosis [11].

In the past several years, the National Institute of Allergy and Infectious Disease (NIAID)/Food Allergy and Anaphylaxis Network (FAAN) and World Allergy Organization (WAO) have published specific clinical criteria to attempt to harmonize the definition of anaphylaxis [12, 13]. Per the 2006 NIAID/FAAN criteria, anaphylaxis is likely if any 1 of the following 3 criteria are met: 1-Acute onset of an illness with involvement of the skin, mucosal tissue, or both and/or respiratory compromise or hypotension or associated symptoms of end organ damage. 2-Two or more of the following occur rapidly after exposure to likely allergen for that patient (minutes to several hours): involvement of the skin mucosal tissue, respiratory compromise, hypotension or associated symptoms, persistent gastrointestinal symptoms. 3- Reduced blood pressure after exposure to a KNOWN allergen for that patient (in infants and children with low systolic blood pressure or greater than 30% decrease in systolic blood pressure or in adults a systolic blood pressure less than 90mmHg or greater than 30% decrease from that person’s baseline) [12].

In 2020, the WAO proposed new criteria to simplify the criteria from NIAD/FAAN. Per WAO 2020 criteria, anaphylaxis is likely when either one of the two criteria are met: Criterion 1-Acute onset of an illness with simultaneous involvement of the skin, mucosal tissue, or both and at least one of the following: respiratory compromise, circulatory compromise, or severe gastrointestinal symptoms. Criterion 2- Acute onset of hypotension, bronchospasm, or laryngeal involvement after exposure to a known or highly probable allergen for that patient, even in the absence of skin findings [13].

There is currently an attempt to create one unified definition between all stakeholders. But for now, most clinicians and researchers use one of the above criteria to determine anaphylaxis.

Based on the above, one can see a dilemma in attempting to diagnose EIA, as one must decide if exercise is a “known” or “likely allergen” or not, as clinical criteria are dependent on this knowledge.

## Exercise Induced Anaphylaxis (EIA)

EIA is defined as anaphylaxis (meeting the criteria above) triggered during or shortly after physical exertion [14]. The prevalence of EIA has been difficult to establish and has only been reported in small case studies that examined anaphylaxis cases only, rather than entire populations. It is estimated to be around 2.3-5% of all anaphylaxis cases reported [7, 8]. In a small case series out of Hong Kong 16 of 29 patients diagnosed with idiopathic anaphylaxis were found to have food dependent exercise induced anaphylaxis (FDEIA) [15]. In a study looking at the incidence of anaphylaxis at an Emergency Department in Spain, 2.4% of cases consistent with anaphylaxis based on NIAID criteria were thought to be exercise induced [16]. EIA occurs across all ages but tends to present in adolescence/young adult hood, affecting males and females equally and tends to be more common in atopic individuals [17, 18].

Establishing the natural course of EIA can be challenging as not only can exercise be the cause of anaphylaxis but exercise is a well-documented co-factor in those with primarily food or drug allergy. In fact, in the European anaphylaxis registry, exercise was associated with severity of anaphylaxis, and in evaluation of only food-induced anaphylaxis, the association held up in peanut-induced anaphylaxis [19, 20]. In a study published by Shaddick et al., 369 patients diagnosed with EIA were followed over 10 years. In their cohort, patients reported initially 14.5 attacks per year but over time most patients have stabilization of symptoms, showing that the frequency of attacks decreased in 47% of subjects and stabilized in 46% of subjects [17]. Most patients experienced reduced attacks by avoiding exercise in temperature extremes (44%), avoiding ingestion of causative foods prior to activity if applicable (37%) and restricting exercise during allergy season (36%) [17].

Several theories have been hypothesized to the underlying mechanism of EIA, including increased gastrointestinal permeability, increase activity of tissue transglutaminase in the gut, redistribution of blood during exercise and mast cell heterogeneity as well as increased plasma osmolality leading to induction of basophil histamine release but none of these theories have been validated to our knowledge to date [18]. Given patients with EIA have been found to have transient elevations in both plasma histamine and serum tryptase [21–24], it does appear that mast cell activation and release of subsequent vasoactive mediators are thought to be responsible for symptoms, just as in other forms of anaphylaxis.

EIA is typically categorized into food-independent and food-dependent (sometimes further divided by the presence of IgE sensitization to the food). Some publications have a category for drug dependent EIA as well [8].

## Food Independent EIA (EIA Alone)

Food independent EIA is where EIA occurs regardless of previous food consumed. EIA is typically reported during times of moderate activity such as jogging, aerobics, walking, bicycling, dancing, and other sporting activities. However, EIA has also been reported during lower intensity activities such as gardening [7, 25]. In general, lower cardiovascular output exercise is thought to be safer [17]. Most report onset of symptoms during brisk activities to occur within 30 min but can occur during any stage of exercise [17]. Interestingly, patients with EIA may not always have symptoms despite repetitive exercise of the same intensity suggesting other co-factors are needed for development of symptoms. Some co-factors may include concomitant illness, extreme temperatures, stress, concomitant allergy pollen season, hormonal changes, NSAID or alcohol use [17].

## Food-Dependent Exercise Induced Anaphylaxis (FDEIA)

FDEIA is defined as anaphylaxis occurring during or shortly following exercise but is dependent upon ingestion of a culprit food, typically within 2 h of the physical exertion although periods of up to 4–6 h have been reported [6, 8]. Typically, a person has IgE sensitization to this food as demonstrated by skin prick testing or serum specific IgE, but not always [26]. In western cultures, the most common culprits include wheat and nuts while in Asian populations wheat and shellfish are most reported [9]. However, a wide array of foods have been reported to induce FDEIA including corn, celery, cow’s milk, mite contaminated wheat flour, tomato, bee pollen, rye and soybean [9, 27–33].

Classically, patients with FDEIA can eat the suspected food (without exercise) and not develop symptoms and conversely can also exercise without symptoms unless the causative food has been ingested within the few hour time frame [9]. Interestingly, however, recent studies have shown that in some patients with FDEIA, anaphylaxis may be triggered even at rest by ingestion of the culprit food if ingested in large quantities, typically much more than in a typical sitting. This would suggest that perhaps some patients with FDEIA simply have food allergy with very high threshold of reaction that goes undetected clinically, and exercise simply lowers the threshold, unmasking the food allergy [23, 26]. To provide plausible explanation for this, it has been hypothesized that gastric permeability increases in exercise, which permits increased entry of intact or incompletely digested allergens into circulation during exercise. This mechanism is similar to how other co-factors of anaphylaxis, such as nonsteroidal anti-inflammatory drugs (NSAID) and alcohol, are thought to act [34, 35]. This mechanism has not been fully proven, however, and in one small study, 12 healthy volunteers undergoing various combinations of aspirin, alcohol, pantoprazole, or exercise did not have increased absorption of gliadin [36]. Additionally, other proposed theories include altered gut microbiome in those with wheat dependent exercise induced anaphylaxis compared to healthy controls and underlying genetic risk-factors as seen in small association studies [37, 38].

Further evidence to support the idea of patients with FDEIA simply having food allergy (and exercise simply acting as a co-factor) has come from data from patients undergoing oral immunotherapy (OIT) to treat food allergies. In most studies of OIT, patients can tolerate a specific dose of the food daily, but then react to that tolerated dose in the presence of exercise [39–42]. Based on this, OIT protocols typically recommend that patients not exercise around the time of dosing their OIT [43, 44]. In addition, there was an elegant study in challenge-proven peanut allergic patients showing exercise could lower threshold of reactivity by ~ 45% as compared to challenge without exercise [45]. 

As stated above, wheat is the most common food associated with FDEIA in both western and Asian countries and to date, is the most studied [9]. The major protein typically implicated is omega-5 gliadin (ω5-gliadin) which is found in the gluten fraction of wheat and is a water-insoluble but ethanol soluble protein. Several studies have shown specific IgE to ω5-gliadin can have a relatively high sensitivity and specificity. In fact, one study by Brockow et al., found that in 16 adults with wheat dependent FDEIA wheat, gluten and ω5-gliadin had a sensitivity and specificity of 81%/87%, 100%/95%, and 100%/97% respectively [34]. Interestingly, there has been some observations that perhaps exercise is not the sole trigger in these patients and that co-factors such as alcohol and NSAIDs can trigger a reaction as well if large amounts of gluten are ingested as some of these patients will react at rest [26, 34, 46].

## Drug Dependent EIA

Drug dependent EIA is a form of EIA that occurs because of co-ingestion of medications and physical exertion. In this form, attacks will not occur if medication ingestion or physical exertion occur independently. The most associated medications are NSAIDs, including aspirin [7]. In studies of patient with history of FDEIA, some patients developed symptoms with the food and aspirin alone, and there appears to be a cumulative effect of exercise and aspirin on reactions in patients with FDEIA [47]. 

**Fig. 1:**
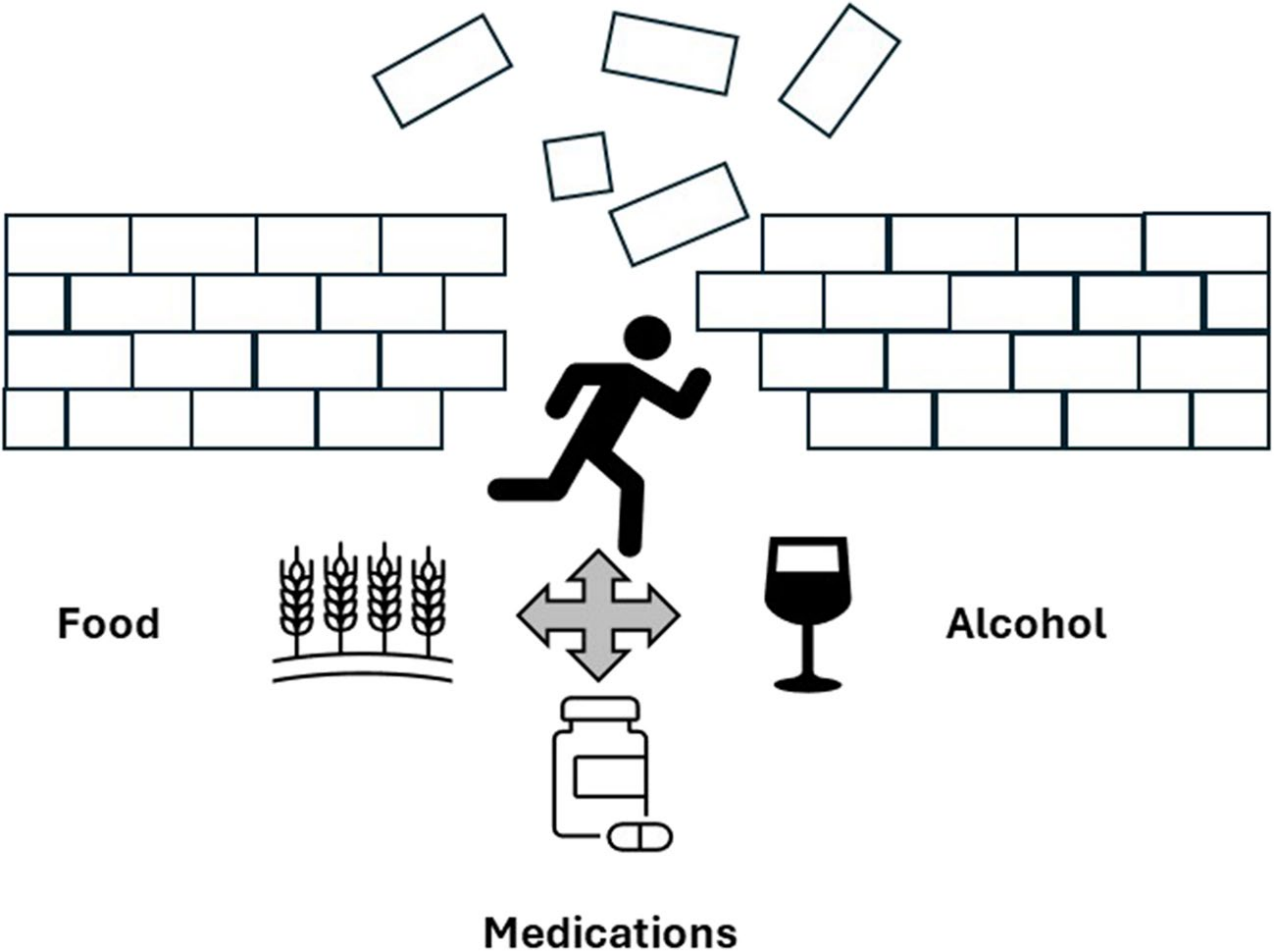
Exercise induced anaphylaxis is a complex interplay of exercise and cofactors such as NSAIDs and alcohol, and many times is dependent on food ingestion

Thus, based on all of the above, it is not fully clear if all of these represent different entities, or if interplay of all of these cofactors lead to reactions in differing phenotypes of similar patients Figure 1.

## Diagnosis

The diagnosis of EIA is largely a clinical diagnosis that relies on a complete history around each, individual anaphylactic episode. EIA should be considered in all patients that present with recurrent episodes consistent with anaphylaxis (which may seem idiopathic). Most patients report episodes beginning with pruritus, flushing/warmth and urticaria during physical exertion prior to progressing into more severe or multiorgan system involvement. In EIA alone, no symptoms will occur without exercise and in FDEIA, the culprit food is ingested typically within 2–3 h of physical exertion. However, on some occasions, symptoms may also occur independent of physical exertion if culprit food is ingested in large quantities or if co-factors such as alcohol ingestion, concomitant use of NSAIDs occur suggesting this particular subset of patients could have primary food allergy with exercise being a co-factor [7, 8].

When gathering a history, it is imperative to ask the type of physical exertion, strenuousness of the activity, duration of the episode, and any interventions that were required for cessation of the episode including possible use of antihistamines or epinephrine. Asking if this tends to occur in certain environments such as in the heat or cold can also provide useful information. Additionally, a complete dietary history can help elicit potential causative foods focusing on the 4–6 h prior to the event. Furthermore, it is very helpful to obtain a list of possible co-factors that could have contributed to the presentation as well including alcohol consumption, use of NSAIDs, recent illnesses, and premenstrual phase of the menstrual cycle in women [7].

When evaluating these patients in the outpatient setting after a presumed episode, the physical exam is often benign. However, patients may have physical features consistent with other atopic conditions such as allergic rhinitis or atopic dermatitis [8]. It is also important to rule out evidence of urticaria pigmentosa or cutaneous mastocytosis as these conditions would also predispose patients to mast cell activation [7].

As part of the work-up for EIA, it is reasonable to obtain a baseline serum tryptase. If the baseline serum tryptase is elevated, this may point to an underlying mast cell disorder such as hereditary alpha tryptasemia (HαT) or systemic mastocytosis [48]. This ideally should be repeated during an acute event to assess for change in baseline serum tryptase. The most studied change in serum tryptase that would suggest mast cell activation is an increase by 2ng/mL + 20% above the baseline value, and if not present, could argue against anaphylaxis [1]. However, this equation has not been validated in EIA.

If one is considering FDEIA, it is imperative to obtain skin testing or serum specific IgE testing to suspected foods because typically a demonstratable sensitization will be noted. Some even consider testing for multiple foods based on the history as on most days patients can tolerate specific foods without reaction so often patients are unlikely to find a specific food as causative. In wheat-suspected FDEIA you can also use IgE to ω5-gliadin as this can be positive even when wheat IgE is negative [15, 23, 34]. Basophil activation testing (BAT) to wheat can also be useful in the diagnosis of wheat dependent EIA if available [49]. However, in a study examining patients on OIT who developed exercise-induced reactions to formerly tolerated OIT doses, BAT could not predict which OIT patients would have these exercise-induced reactions [50]. 

One can also consider exercise challenge testing as this has been found to have varying success rates and there are few protocols regarding how to administer the testing [3, 26, 28, 34]. Some studies have used protocols where patients use a treadmill at varying increase in speeds using the Bruce protocol for 30 min with spirometry being utilized as well as obtaining vital signs including blood pressure to see if a response is elicited [3]. If a challenge is positive in the absence of suspected food ingestion, this is diagnostic of EIA alone. However, if exercise challenge is negative this does not necessarily exclude the diagnosis and one could consider food/exercise challenges as well as eliciting with co-factors such as NSAID use by administering aspirin prior to the challenge as utilized by Brokow et al. [34].

## Differential Diagnosis

Often EIA can be difficult to diagnose as can present similarly to other known conditions. For example, in cholinergic urticaria, a patient can develop punctate urticarial wheals from exercise but would also experience symptoms with any means of passively heating core body temperature including hot showers or use of a sauna. Additionally, cholinergic urticaria can also have generalized symptoms if there is enough cutaneous mast cell release [7]. Some patients will also experience cold-induced urticaria that can cause sufficient systemic mediator release leading to anaphylaxis. This can be confusing as some patients exercising in cold weather may be diagnosed with EIA when symptoms are most likely cold-induced urticaria. This can be diagnosed by use of an ice cube test where passive cooling should provoke symptoms [51]. Other forms of urticaria such as physical urticaria or acute spontaneous urticaria can occur during exercise making EIA on the differential; however, episodes should also occur outside of exercise and would not have systemic features. Other diagnosis such as underlying mast cell disorders, food allergies, NSAID related anaphylaxis can also be seen during periods of exercise. In fact, cardiovascular events can also be misdiagnosed as EIA as can present similarly [8] (Table 1).

**Table 1:** Differential diagnosis of patients presenting with signs and symptoms concerning for EIA and potential ways to differentiate EIA from other conditions

**Diagnosis**	**Possible ways to differentiate from EIA**
Cholinergic Urticaria	Typically smaller, punctate wheals. Occur with passive forms of heating.
Cold-Induced Urticaria	Symptoms occur outside of physical activity and can have systemic features. Can consider ice cube test to aid in diagnosis.
Acute or Chronic Spontaneous Urticaria	Urticarial lesions will typically occur in isolation regardless of physical activity.
Underlying mast cell disorder (systemic mastocytosis, hereditary alpha tryptesemia, mast cell activation syndrome)	Episodes consistent with mast cell activation that could appear consistent with anaphylaxis but would also occur in various settings. Elevated baseline tryptase could be helpful in differentiating these.
Food allergy alone	Symptoms occur upon ingestion of causative food regardless of recent physical activity
NSAID allergy	Symptoms occur upon ingestion of causative medication regardless of recent physical activity
Cardiovascular syndrome (arrhythmia, syncopal event).	Typically, no skin findings and usually would see other vital sign changes such as hypotension or tachycardia.

## Management

The goal of therapy in EIA is to minimize the frequency and severity of future reactions. Every patient with known diagnosis of EIA should carry an epinephrine auto-injector and be able to recognize the signs and symptoms of anaphylaxis. Additionally, management of EIA should involve shared-decision making and can be personally tailored to each patient, as many patients may wish to continue to exercise in some capacity.

A first-line approach could be starting with low-level activity and increasing as tolerated. It is also recommended to either exercise in a supervised setting, with a partner, or with access to a device which can alert for help if needed (e.g. a smart watch). In those with FDEIA, avoiding the culprit food for approximately 4 h before and one hour after activity would be recommended [46]. It would also be reasonable to avoid exercise while any of a patient’s known co-factors (alcohol, NSAID use, menstrual cycle related issues, illness, extreme temperatures, or specific allergy season) are present as these are known to decrease a person’s reaction threshold [26, 34]. Lastly, encouraging patients to stop activity at the first sign of symptoms such as pruritus, flushing, or urticaria.

If patients can modify behavior with above measures, pharmacotherapy is not necessary. However, in patients where this approach is not feasible, one can consider prophylactic medications with the understanding that these medications will not prevent anaphylaxis but can reduce mild symptoms such as urticaria and angioedema. The use of H1 antihistamines as premedication has not been well studied and there are concerns about masking early symptoms; however, if a patient predominantly has cutaneous findings only this is not unreasonable [17, 46, 52]. If used, non-sedating antihistamines are preferred at once or twice daily dosing. In small numbers of case reports, the adjuvant use of Montelukast has been shown to reduce symptoms as well [53]. In patients with FDEIA that have difficulty avoiding culprit foods, high-dose cromolyn has been reported to potentially prevent an attack may also be useful in preventing an attack if taken prior to culprit food being ingested [54]. Misoprostol has shown reported benefit in case reports. Finally, consideration of omalizumab has been shown as effective in refractory cases [55–58].

## Future Directions

Better diagnostic strategies are needed for the accurate diagnosis of EIA and its varied clinical presentations. Validated exercise challenge protocols and additional testing modalities for suspected foods could lead to improved diagnostic utility and more precise treatment plans for patients. For patients with FDEIA, current investigational therapies include sublingual immunotherapy using high gluten flour that could potentially increase reaction threshold in those patients as well as production of wheat flours that have reduced immunologic potential [59, 60].

## Conclusion

EIA, historically defined as either food independent or food dependent, is a complex condition that can be difficult to diagnose and manage due to the various effects of co-factors on the clinical presentation. In fact, in some patients it can be difficult to determine whether one has true EIA, primarily a food or drug allergy that is manifested with a co-factor such as exercise, or an alternative diagnosis that can have features mimicking EIA. Although there is no standardized, confirmatory testing for EIA, understanding the complex interplay of various co-factors on these patients can aid the clinician in appropriate diagnosis as well as developing a personalized treatment plan to ensure a meaningful quality of life for the patient.

## Data Availability

No datasets were generated or analysed during the current study.
